# Establishment of reference intervals of thyroid function tests from cord blood of neonates in two selected hospitals, Addis Ababa, Ethiopia

**DOI:** 10.1186/s12887-016-0654-2

**Published:** 2016-08-02

**Authors:** Aman Mehari, Feyssa Challa, Goitom Gebreyesus, Dereje Alemayehu, Daniel Seifu

**Affiliations:** 1 Department of Medical Biochemistry and Molecular Biology, Biomedical Institute, College of Health Sciences, Mekelle University, Mekelle, Ethiopia; 2 Clinical Chemistry Department, Directorate of TB & HIV research, Ethiopian Public Health Institute, Addis Ababa, Ethiopia; 3 Department of Pediatrics, College of Health Sciences, Addis Ababa University, Addis Ababa, Ethiopia; 4 Department of Pediatrics, Gandhi Memorial Hospital, Addis Ababa, Ethiopia; 5 Department of Biochemistry, College of Health Sciences, Addis Ababa University, Addis Ababa, Ethiopia

**Keywords:** Thyroid function tests, Reference intervals, Screening program, Congenital hypothyroidism

## Abstract

**Background:**

Reference intervals are affected by different factors such as lifestyle, ethnicity, age/developmental stage, gender, nutrition and other environmental factors (Clin Biochem Rev: 29,2008). Therefore, it is obvious that it should be established for every population in different regions even within a country. Then the aim of this study is to establish population specific reference intervals of thyroid stimulating hormone, free thyroxine and free triidothyronine levels of cord blood.

**Results:**

One hundred twenty three cord blood samples collected from the umbilical cord of newborns were analyzed for thyroid stimulating hormone, free thyroxine and free triidothyronine values. The birth weights ranged between 2500 and 4700 g with mean (SD) value of 3241.46 (459.495) gram. Their gestational age ranged between 37 and 44 weeks with an average of 39.74 weeks. The 2.5^th^ and 97.5^th^ percentiles of values were found to be 3.48 mIU/L and 27.57 mIU/L for thyroid stimulating hormone, 0.89 ng/dl and 1.53 ng/dl for free thyroxine and 1.19 pg/ml and 2.51 pg/ml for free triidothyronine respectively.

**Conclusion:**

In the present study the reference intervals of thyroid stimulating hormone, free thyroxine and free triidothyronine were established and based on the results obtained, were 3.48–27.56 mIU/L for thyroid stimulating hormone, 0.89–1.53 ng/dl for free thyroxine and 1.19–2.51 pg/ml for free triidothyronine. It has been concluded that the result can provide us with an important baseline to establish population specific reference intervals for our country using large scale studies.

**Electronic supplementary material:**

The online version of this article (doi:10.1186/s12887-016-0654-2) contains supplementary material, which is available to authorized users.

## Background

The development and maturation of the brain and central nervous system and other target tissues have a critical dependence on thyroid hormones, beginning before birth and extending through the first 2–3 years of life. Therefore, abnormal thyroid function may lead to different developmental problems. Mental retardation due to congenital hypothyroidism is one of the serious consequences of thyroid abnormalities. The incidence of congenital hypothyroidism ranges from 1 in 3000 to 1 in 4000 newborn infants and is a common preventable cause of mental retardation [1]. Thyroid gland secretes two major hormones, thyroxine and triiodothyronine, commonly called T4 and T3, respectively [2]. Thyroid hormone production is regulated by another hormone called thyroid-stimulating hormone (TSH). TSH is made by the pituitary gland, which is located in the brain. From the pituitary gland, TSH travels to the thyroid where it stimulates the production of T3 and T4 and their release into the bloodstream [3, 4]. Thyroid function tests are used to evaluate the thyroid’s functioning and to diagnose and help determine the cause of thyroid diseases.

Since reference intervals are affected by different factors such as lifestyle, ethnicity, age/developmental stage, gender, nutrition and other environmental factors [5], it is obvious that it should be established for every population in different regions, even within a country. However, there is no implemented population specific reference intervals for almost all analysts, including the thyroid function tests in Ethiopia, let alone for regional areas. Current pediatric reference intervals for TSH, T4, and T3 have been derived predominantly from samples collected on hospitalized infants and children of the western population [5], and may not reflect levels in healthy multicultural populations. Application of reference ranges, specific to other ethnic groups is clinically inappropriate for many biomarkers including thyroid function tests. Another problem with the available pediatric reference intervals, is that they were determined over two decades ago using older/less accurate instrumentation and methodologies that are no longer relevant to testing technology used by clinical laboratories today [6, 7].

Since the 1970’s, neonatal screening programs for hypothyroidism have been developed and have become popular worldwide. Specimens from cord blood and filter paper spotted with blood from a heel break can be utilized to measure TSH and fT4 for Congenital hypothyroidism screening. Due to early discharges from hospitals in Ethiopia, it is usually difficult to take blood samples from neonates after a few days of life [8]. Therefore, cord blood is the specimen of choice as it is readily available at birth, with adequate volume for supplemental tests when required. Therefore, the cord blood specimen is selected in our study with special interest of the study group to set an indispensable prerequisite of neonatal screening program for congenital hypothyroidism which is part of the postnatal care in the developed countries. It has been concluded that the result can provide us with an important baseline to establish population specific reference intervals for our country using large scale studies.

## Methods

A cross sectional study was conducted from July, 2013 to January, 2013 using cord blood sample from 123 newborn infants of both sexes. The study involves consecutive births of newborn infants from Tikur Anbessa Hospital and Gandhi Memorial Hospital selected using a set of inclusion and exclusion criteria with the help of experienced pediatricians and Gynecologist.

Healthy, full term and singleton newborns were included and preterm, multiple births and infants with evidence of infection were excluded from the study. A convenient sampling technique was used to take 138 study participants. Sample size determination was, according to recommendations of international organizations which states that the preferred method as a priori nonparametric determination of reference intervals of the single group population should be established at least from 120 reference individuals. In this study 138 samples were taken with 10 % contingency plan for non respondents and participants who may excluded after blood collection. The Clinical Laboratory Standards Institute (CLSI) and International Federation of Clinical Chemistry and laboratory medicine (IFCC) were the main references to decide the sample size of 120. According to those international organizations 120 is the minimum sample size required to determine reference intervals for the 95^th^ percentile reference limits (2.5^th^ and 97.5^th^ percentile).

Five ml Cord blood samples were collected immediately after birth from the umbilical cord of the neonates of volunteer parent. Cord blood was obtained after an uncomplicated vaginal delivery and the umbilical cord was ligated from the placental side. BD vacutainer EDTA tubes were used to collect blood sample and the plasma was separated as soon as possible through centrifugation at 4600 rpm for 4 min. Plasma samples were stored in a deep freezer at −20 °C in the department of Biochemistry laboratory until it transports to Ethiopian Health and Nutrition Research Institute (EHNRI). Finally the samples were transported using the cold box and stored in a deep freezer at −80 °C in EHNRI Clinical Chemistry Laboratory until the hormone analysis was done.

### Assays of laboratory variables

TSH, fT4 and fT3 were measured using Elecsys 2010 immunoassay analyzer (**Cobas®**) which is a fully automatic run-oriented analyzer system for the determination of immunological tests using the electrochemiluminescence immunoassay “ECLIA” process. All components and reagents for routine analysis are integrated in or on the analyzer. Specific test principles of the hormones to be measured are explained as follows.

### TSH assay

Sandwich immunoassay with total duration of assay of 18 min was used. It has two step incubation periods. During the first incubation period 50 μL of samples, a biotinylated monoclonal TSH‑specific antibody and a monoclonal TSH‑specific antibody labeled with a ruthenium complex react to form a sandwich complex. At the second incubation streptavidin-coated microparticles was added and the complex becomes bound to the solid phase via interaction of biotin and streptavidin. The reaction mixture was aspirated into the measuring cell where the microparticles are magnetically captured onto the surface of the electrode. Unbound substances, then removed with ProCell/ProCell M. Application of a voltage to the electrode induces chemiluminescent emission which was measured by a photomultiplier.

### fT3 assay

A competitive immunoassay system with total duration of assay of 18 min was used. It has two step incubation periods. During the first incubation period 15 μL of samples and an anti‑T3‑specific antibody labeled with a ruthenium complex was mixed. And the second incubation period starts with the addition of biotinylated T3 and streptavidin-coated microparticles, and the still‑free binding sites of the labeled antibody become occupied, with formation of an antibody‑hapten complex. The entire complex binds to the solid phase via interaction of biotin and streptavidin. The reaction mixture, then aspirated into the measuring cell where the microparticles are magnetically captured onto the surface of the electrode. Unbound substances were removed with ProCell/ProCell M. Application of a voltage to the electrode then induces chemiluminescent emission which was measured by a photomultiplier.

### fT4 assay

A competition immunoassay system with total duration of assay of 18 min was used. It has two step incubation periods. During the first incubation period 15 μL of samples and an anti‑T4‑specific antibody labeled with a ruthenium complex was mixed. And the second incubation period starts with the addition of biotinylated T4 and streptavidin-coated microparticles, and the still-free binding sites of the labeled antibody become occupied, with formation of an antibody-hapten complex. The entire complex was bounded to the solid phase via interaction of biotin and streptavidin. The reaction mixture, then aspirated into the measuring cell where the microparticles are magnetically captured onto the surface of the electrode. Unbound substances were removed with ProCell/ProCell M. Application of a voltage to the electrode then induces chemiluminescent emission which was measured by a photomultiplier.

Results of all of the hormones of thyroid functions test TSH, FT4, FT3 were determined via a calibration curve, which is instrumental-specifically generated by 2-point calibration and a master curve provided via the reagent barcode.

### Data management and statistical analysis

Data was analyzed using the SPSS version 20. Prior to the calculation of reference intervals, data were cleaned. Records with missing descriptive information and outliers were removed before statistical analysis. Outliers were excluded using the following formulas which are suggested by most literatures.

Outlier excluding formula:

$$ \begin{array}{c}\hfill \mathrm{Upper}\ \mathrm{Limit} = {\mathrm{Q}}_3+\left(2.2\ *\ \left({\mathrm{Q}}_3\hbox{-} {\mathrm{Q}}_1\right)\right.\hfill \\ {}\hfill \mathrm{Lower}\ \mathrm{Limit} = {\mathrm{Q}}_1\hbox{-} \left(2.2*\ \left({\mathrm{Q}}_3\hbox{-} {\mathrm{Q}}_1\right)\right.\hfill \\ {}\hfill \mathrm{Where}\ \mathrm{Q}\ \mathrm{stands}\ \mathrm{f}\mathrm{o}\mathrm{r}\ \mathrm{Q}\mathrm{uartile}\hfill \end{array} $$

Once the upper and lower limits are obtained, results higher than the upper limit and lower than the lower limit were removed from the data. A net of 123 participants were included for obtaining the reference intervals. It should be noted that outliers were not excluded from all data analysis. The effects of maternal age, gestational age, gender and birth weight on newborn TSH, FT4 and FT3 levels were assessed by analysis of variance (ANOVA), multiple correlation coefficient and Spearman’s correlation coefficients. Multiple linear regressions were performed to quantify the associations between the above parameters and changes in TSH, FT4 and FT3 levels. Histogram and Q-Q plot for visual and Kolmogorov-Smirnove (K-S) Test for numeric assessment of the distribution of data were used. A probability level of *p* < 0.05 was taken as significant.

Since the numerical values of TSH do not have normal Gaussian distributions, log transformation was required and the non-parametric method was used which is recommended by IFCC and CLSI for such type of data with a minimum of 120 individuals required per partition. Reference limits were calculated using non-parametric ascending rank order statistics. The conventional 95^th^ percentile reference limits were determined by calculating the rank numbers for the 2.5^th^ and 97.5^th^ percentiles.

### Quality control and quality assurance

The quality of the results was maintained by following the standard operational procedures for analysis of TSH, fT4 and fT3 prepared by the senior staffs of EHNRI clinical chemistry Laboratory. The Normal and pathological controls, Preci control Universal, Roche, 11731416122 PC U1 and PC U2 were also performed with every run to control the performance of the overall procedure and the instrument under use. The specified laboratory is currently participating in international quality assurance programs which make our result more reliable.

## Results

**Fig. 1:**
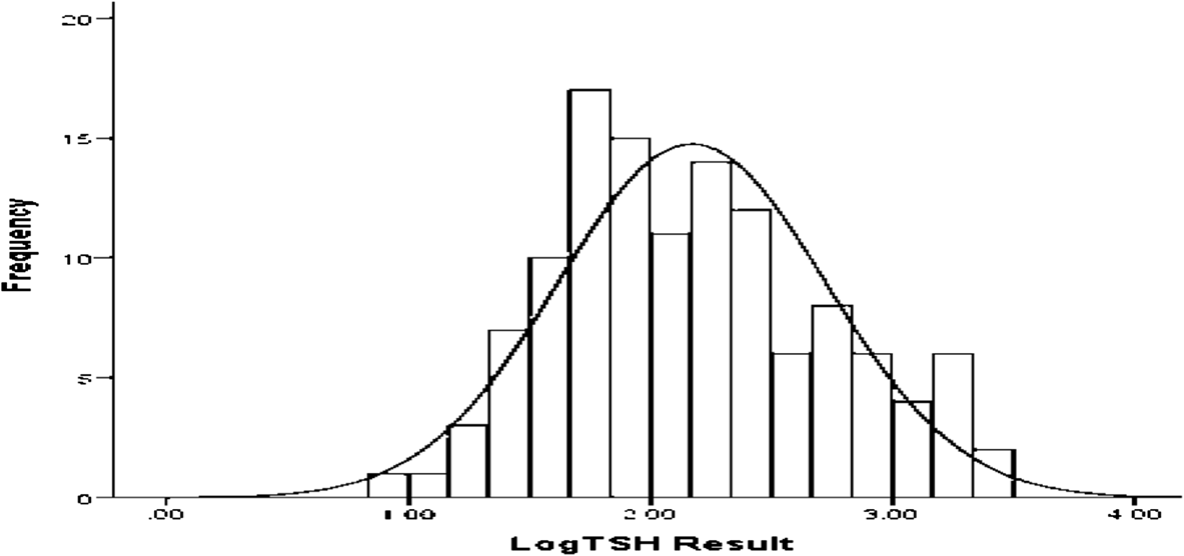
Frequency distribution of maternal age of the study participants

**Fig. 2:**
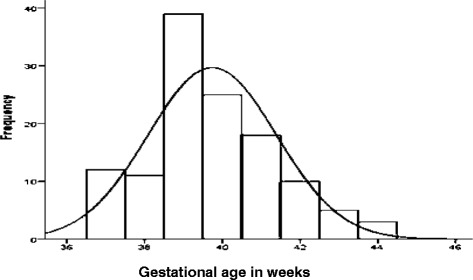
Frequency distribution of Gestational age of study participants

One hundred twenty three cord blood samples collected from umbilical cord of newborns with male to female ratio of 0.83 (56 males and 67 females) were analyzed for TSH, fT4 and fT3 values. Their birth weight ranged between 2500 g and 4700 g with mean (SD) value of 3241.46 (459.495) gram and 47 (38.8 %) of them have birth weight in the range of 3000–3490 g. Table 1 presents the distribution of study participants in ranges of birth weight. Their gestational age ranged between 37 and 44 weeks. 74.8 % of them were born in the range of 39–42 weeks of gestational age. Table 2 shows the Gestational age distribution of the newborns and Fig. 2 presented it in the histogram. The maternal age is displayed in Fig. 1 in which 82.9 % of them were under the age of 30 years.

**Table 1:** Birth weight wise distribution of samples

Birth Weight in gram	Number of Samples (% ages)
	*N* = 121
2000–2990	30 (24.8)
3000–3490	47 (38.8)
3500–3990	36 (29.8)
>4000	8 (6.6)

**Table 2:** Distribution of study participants in ranges of Gestational ages

Gestational age in weeks	Number of samples (% ages)
37–38	23 (18.7)
39–42	92 (74.8)
>42	8 (6.5)
Total	123

**Fig. 3:**
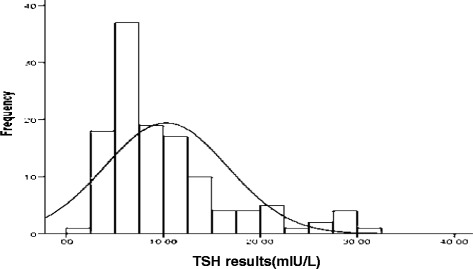
Distribution of TSH results among all the study participants

The conventional 95^th^ percentile reference limits (2.5^th^ and 97.5^th^ percentile) method was used to determine the reference ranges of TSH, fT4 and fT3 values of the cord blood. Prior the establishing of the reference intervals the data were tested for normal distribution. The overall distribution of TSH result was positively skewed (Fig. 3), with skewness and kurtosis of 1.41 and 1.46 respectively. TSH values were further tested for normal distribution using Kolmogorov-Smirnove (K-S = 0.007) test and were not normally distributed. Based on the K-S test result obtained the lognormalization was necessary. After logarithmically normalized (Fig. 1) and 2.5^th^ and 95.7^th^ percentile values were computed.

**Fig. 4:**
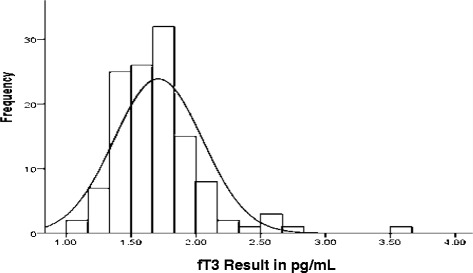
Frequency distribution of fT3 results

Additionally it founds that there was no correlation of cord blood values of thyroid function tests with maternal age, gestational age, birth weight and sex.

### TSH results

The Minimum and Maximum values after exclusion of outliers was 2.40 mIU/L and 30.15 mIU/L respectively. The mean (SD) and median values were 10.27 (6.31) and 8.37 mIU/L respectively. The 2.5^th^ and 97.5^th^ percentiles of TSH values were found to be 3.48 mIU/L and 27.56 mIU/L respectively (Additional file 1). The combined and separate mean values for both sexes displayed in Table 3. Table 4 depicts the TSH values of the entire participants.

**Table 3:** Mean values of cord blood TSH, fT4 and fT3 in male and female newborns

Variables	Mean ± SD males	Mean ± SD females	Mean ± SD Total	*P*-value
TSH (mIU/L)	11.17 ± 6.6	9.52 ± 6	10.27 ± 6.31	0.151
fT4 (ng/dL)	1.16 ± 0.14	1.19 ± 0.14	1.17 ± 0.14	0.195
fT3 (pg/mL)	1.67 ± 0.28	1.73 ± 0.34	1.70 ± .034	0.302

**Table 4:** Summary of umbilical cord blood TSH levels

TSH result in mIU/L	No of Samples (% ages)
<4	6 (4.9)
4–7.99	52 (42.3)
8–11.99	33 (26.8)
12–15.99	13 (10.6)
16–19.99	6 (4.9)
20–24.99	6 (4.9)
25–29.99	6 (4.9)
30–34.99	1 (0.8)
Total	123

**Table 5:** 2.5^th^ and 97.5^th^ percentiles of TSH, fT4 and fT3 values

Variables	Prior to log normalized		Log normalized	
	2.5^th^	97.5^th^	2.5^th^	97.5^th^
TSH (mIU/L)	3.48	27.57	3.48	27.56
fT4 (ng/dL)	.89	1.53	0.89	1.53
fT3 (pg/mL)	1.19	2.51	1.19	2.51

### fT4 results

The Minimum and Maximum values of fT4 were 0.71 ng/dL and 1.57 ng/dL with mean (SD) and median values of 1.17 (0.14) and 1.17 ng/dL respectively. 1.16 ng/dL and 1.19 ng/dL were the mean values for males and females respectively. The 2.5^th^ and 97.5^th^ percentile were 0.89 ng/dL and 1.53 ng/dL (Table 5) (Additional file 1).

### fT3 results

The Minimum and Maximum values of fT3 were 1.08 pg/mL and 3.66 pg/mL with mean (SD) and median values 1.70 (0.34) and 1.67 pg/mL respectively. The 2.5^th^ and 97.5^th^ percentiles were 1.19 pg/mL and 2.51 pg/mL respectively (Additional file 1).

## Discussion

Congenital hypothyroidism often causes irreversible mental retardation if thyroid hormone replacement therapy is not begun during the first few months of life. The successful introduction of screening in the 1970’s has enabled North America, Europe, to a limited extent Asia, Latin America and a few African countries to combat the ill effects of Congenital hypothyroidism and saved lives. Those screening programs have successfully helped in early diagnosis and treatment of congenital hypothyroidism [9, 10]. It has not been able to implement in Ethiopia because of several factors, like cost, lack of reliable laboratories on a large scale, and unavailability of baseline data to our population. The use of cord blood TSH or combined with fT4 as a screening tool is an attractive proposition because of its simplicity and accessibility. Although several investigators have measured TSH and T4 in a cord and serum samples from both premature and term infants, every reference laboratory needs to establish its own normal values in order to validate its own data and technical expertise. Population-specific reference intervals are an important prerequisite for interpreting thyroid hormone measurements. In addition to that, the clinical value of TSH, free thyroxine and free triiodothyronine analysis depends on the reference intervals with which they are compared [5]. Therefore, it is important to have population specific normal values for this age group to avoid misdiagnosis and incorrect treatment. The present study was undertaken to establish standard reference values for better evaluation of thyroid function in cord sera in selected hospitals, Addis Ababa, Ethiopia.

Values of TSH analysis is summarized in Table 4 and it shows a comparable trend with other reports by various investigators across the globe. 74.0 % of the cord blood results are less than 12 mIU/L, slightly higher than reports from India, where 85.75 % of results found to be less than 12 mIU/L [11, 12]. Comparison of means of both sexes was performed using one way ANOVA. Based on the results obtained from this investigation, there is no statistically significant difference between the values for males and females in TSH (*p value 0.151,*), fT4 (*p value 0.195*) and fT3 (*p value 0.302*). This implies that there is no need to establish gender specific reference intervals.

Previously, few studies have reported in establishing cutoff values of cord blood TSH in Ethiopia. One study from yalemtsehay and co-workers was performed, at the same study area with the current study, and they determined the cutoff value of cord blood TSH (the 97.8^th^ percentile in this case) was 15.4 mIU/L [8]. Another study, which was not on the cord blood sample, had measured TSH values of blood from a heel-pricks in neonates from six hours of life up to seven days old infants. They determined the cut- off point for TSH to be 29.4 mIU/l [13, 14]. In the present work the 97.5^th^ percentile of cord blood TSH was 27.56mIU/L which shows higher than reports from yalemtsehay and co-workers. This can be explained by the difference in method of analysis. Immunoradiometric assay was used in the previous study which had low sensitivity as 16.1 % of males and 30.5 % of females were reported for undetected results [8] whereas the lowest values was 2.40 mIU/L in the present study using the Electrochemiluminescence Immunoassay Method. Other possible explanations were the previous study includes all the preterm and full term infants which can lower the cut-off values of TSH. Whereas only full term infants were included in the present work as if it had small sample sizes relative to the mentioned study.

Many studies reported that cord blood TSH can be used as a screening tool for congenital hypothyroidism from all over the world. A study from Japan had shown that mixed cord blood is a good sampling technique for screening of congenital hypothyroidism [15–17]. And it was concluded that cord TSH had a better specificity and sensitivity as compared to cord or filter paper T4 at 3–5 days of age. A study from Iran shows the reference range of TSH concentration ranged from 0.77 to 24.91mlU/mL with a mean value of 7.09 [18, 19] which had lower values comparing to the present study. However, the time of establishment shows it was done with older methods which may have lower sensitivity. This implies us the importance of method specific and timely updated reference values. One study from India reported the 97^th^ percentile result of TSH as 25.8 mIU/L [11] which was comparable but lower than the result of the present study. Both of the above studies had lower cut-off values of TSH relative to the current result obtained which can be explained by the timing of the research as old methods were used and the difference in the target population.

**Fig. 5:**
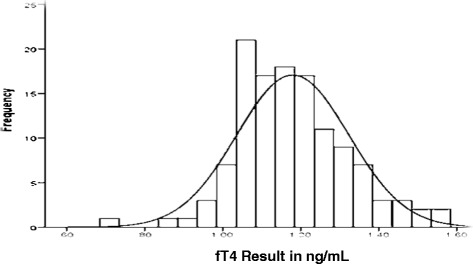
Distribution of fT4 result among all study participants

No study was conducted before with the issue of establishing reference ranges for the cord blood values for fT4 and fT3 in Ethiopia. Possibly the result of this study could be the first population specific base line data. The 2.5^th^ and 97.5^th^ percentiles are 0.89 ng/dL and 1.53 ng/dL for fT4 and 1.19 pg/mL and 2.51 pg/mL for fT3 respectively. Figure 5 presents the distribution of fT4 result among all study participants. The data were slightly skewed with skewness and kutosis value 0.262 and of 0.679 respectively but K-S test result shows fT4 values were normally distributed. Figure 4 presents the distribution of fT3 result among all study participants. And the data was slightly skewed data with skewness and kurtosis values of 1.973 and 8.24 respectively. K-S test result indicates fT3 results were normally distributed but have a close test value with the level of significance (0.053). The results of test of normally distribution for fT4 and fT3 shows that result were normally distributed. Therefore, the reference ranges were calculated directly from the untransformed data. The results were compared with studies from abroad and were comparable but slightly lower than results of one study from Turkey as it reported the 2.5^th^ and 97.5^th^ percentiles to be 1.07 ng/dL and 2.02 ng/dL for fT4 and 1.15 pg/mL and 2.81 pg/mL for fT3 [9].

TSH, fT4 and fT3 value were tested using the nonparametric test, kruska - wallis test, of correlation with Maternal age, Birth weight and Gestational age. Despite the reports which concluded that Maternal age and Gestational age were related to neonatal TSH level and another study showed that Gestational age was independently associated with lower cord TSH and high TT4 [20, 21], there was no statistically significant association of the variables with any of the listed maternal and fetal factors.

## Conclusion

The study was conducted to estabilish reference intervals of TSH, FT4 and FT3 in cord blood of newborns. The results obtained were 3.48 - 27.56 mIU/L for TSH, 0.89 - 1.53 ng/dL for fT4 and 1.19 - 2.51 pg/mL for fT3. Even though setting of cut-off values need its own expertise panel discussion, based on the reference range of cord TSH obtained in this study, it is safe to use the conventional cut-off value of TSH, 20mIU/L, for recall purpose in the target population.

The present study, similar to other reports, suggests that TSH levels in cord blood might be a feasible alternative specimen for a congenital hypothyroidism screening program in countries where neonatal blood is not easily attainable. In the given situation of Ethiopia, where hospital discharge is within about 24 h [6], using cord blood samples has to be practiced for screening purpose of congenital hypothyroidism, given all other necessary infrastructures put in place. It was confirmed by other studies, that cord blood could be used to prepare blood spots and the present study adds cut-off values using improved method, this shows an excellent progress towards launching the screening program in our country.

## Abbreviations

CLSI, Clinical Laboratory Standards Institute; ECLIA, electrochemiluminescence immunoassay; EHNRI, Ethiopian Health and Nutrition Research Institute; FT3, free triiodothyronine; FT4, free thyroxine; IFCC, International Federation of Clinical Chemistry; TSH, thyroid-stimulating hormone; TT3, total triiodothyronine; TT4, total thyroxine

## Additional file

Additional file 1: TSH, fT4 and fT3 results of Study participants. (XLSX 15 kb)

## References

[1] Mockule I, et al. The newborn thyroid stimulating hormone levels in relation to maternal age and gestation at birth. Lithuania obstetrics and gynecology, 2010;XIII:No. 3.

[2] C. Guyton and Hall (2006). Text Book of Medical Physiology.

[3] Boyd JC (2010). Defining laboratory reference values and decision limits populations, intervals, and interpretations. Asian J Androl.

[4] Zhang J and Lazar MA. The mechanism of action of thyroid hormones. 2011. 10.1146/annurev.physiol.62.1.439 10845098

[5] Schnabl K, Chan MK, Gong Y, Adeli K. Closing the Gaps in Paediatric Reference Intervals. The CALIPER Initiative. Clin Biochem Rev. 2008;29:89–96. PMC2605413 19107221

[6] Delvin EE, Laxmi Grey V, Vergee Z (2006). Gap analysis of pediatric reference intervals related to thyroid hormones and the growth hormone–insulin growth factor axis. Clin Biochem.

[7] Kratzsch J, Schubert G, Pulzer F, Pfaeffle R, Koerner A, Dietz A, Rauh M, Kiess W, Thiery J (2010). Reference intervals for TSH and thyroid hormones are mainly affected by age, body mass index and number of blood leucocytes, but hardly by gender and thyroid autoantibodies during the first decades of life. Clin Biochem.

[8] Mekonnen Y, Hawariat GW, Chamiso B, Raue F (2004). Thyroid Stimulating Hormone values from cord blood in neonates. Ethiop J Health Dev.

[9] Mutlu M. et al. Reference intervals for thyrotropin and thyroid hormones and ultrasonographic thyroid volume during the neonatal period in Turkey. J Matern Fetal and Neonatal Med, UK. Ltd. 2011. doi:10.3109/14767058..561894 10.3109/14767058.2011.561894 21410423

[10] Adachi M, Soneda A, Asakura Y, Muroya K, Yamagami Y, Hirahara F (2012). Mass screening of newborns for congenital hypothyroidism of central origin by free thyroxine measurement of blood samples on filter paper. Eur J Endocrinol.

[11] Manglik AK, Chatterjee N and Ghosh G. Umbilical Cord Blood TSH Levels in Term Neonates: A Screening Tool for Congenital Hypothyroidism. East A Medical J. 2000;77(7):377–81. 16269841

[12] Mc Elduff A, McElduff P, Wiley V, Wilcken B (2005). Neonatal thyrotropin as measured in a congenital hypothyroidism screening program. Influence of mode of delivery. J Clin Endocrinol Metab.

[13] Feleke Y, Enquoselassie F, Deneke F, Abdulkadir J, Hawariat GW, Tilahun M, Mekbib T. Neonatal congenital hypothyroidism screening in Addis Ababa, Ethiopia. Indian Pediatr.2005;42(10):1029–32. 12862157

[14] Wassie E, Abdulkadir J (1990). Normal thyroid function values in Ethiopians. Ethiop Med J.

[15] Fuse Y, Wakae E, Nemoto Y, Uga N, Tanaka M, Maeda M (1991). Influence of perinatal factors and sampling methods on TSH and thyroid hormone levels in cord blood. Endocrinol Jpn.

[16] Özön ZA (2008). Congenital hypothyroidism monitorization programs, current status in Turkey Turkiye Klinikleri. J Endocrin Spec Top.

[17] Soldin OP, Jang M, Guo T, Soldin SJ. Pediatric Reference Intervals for Free Thyroxine and Free Triiodothyronine. Mary Ann Liebert, Inc. 2009. doi:10.1186/1089=thy.0037. 10.1089/thy.2009.0037 PMC2875983 19583487

[18] Williams FL, Ogston SA, van Toor H (2005). Serum thyroid hormones in preterm infants: associations with postnatal illnesses and drug usage. J Clin Endocrinol Metab.

[19] Karamizadeh Z and Amirhakimi GH. Thyroxine and thyrotropin levels in cord blood sera from south of iran. Med J Islam Repub Iran. 1997;11:133–135.

[20] Herbstman J, Apelberg BJ, Witter FR, Panny S, Goldman LR (2008). Maternal, infant, and delivery factors associated with neonatal thyroid hormone status. Thyroid.

[21] Feingold SB, Brown RS, Stanton BF (2010). Neonatal Thyroid Function, NeoReviews;11;e640-e646. Nelson textbook of pediatrics.

